# Non-Destructive Current Sensing for Energy Efficiency Monitoring in Buildings with Environmental Certification

**DOI:** 10.3390/s150716740

**Published:** 2015-07-10

**Authors:** Lia Toledo Moreira Mota, Alexandre de Assis Mota, Lorenzo Campos Coiado

**Affiliations:** Pontifical Catholic University of Campinas, CEATEC, Campus I, Rod. Dom Pedro I, Km136, CEP 13086-900, Campinas, São Paulo, Brazil; E-Mails: lia.mota@puc-campinas.edu.br (L.T.M.M.); lorenzo.coiado@gmail.com (L.C.C.)

**Keywords:** non-destructive method, current sensor, energy efficiency, energy consumption, air-core transformer, building certification

## Abstract

Nowadays, buildings environmental certifications encourage the implementation of initiatives aiming to increase energy efficiency in buildings. In these certification systems, increased energy efficiency arising from such initiatives must be demonstrated. Thus, a challenge to be faced is how to check the increase in energy efficiency related to each of the employed initiatives without a considerable building retrofit. In this context, this work presents a non-destructive method for electric current sensing to assess implemented initiatives to increase energy efficiency in buildings with environmental certification. This method proposes the use of a sensor that can be installed directly in the low voltage electrical circuit conductors that are powering the initiative under evaluation, without the need for reforms that result in significant costs, repair, and maintenance. The proposed sensor consists of three elements: an air-core transformer current sensor, an amplifying/filtering stage, and a microprocessor. A prototype of the proposed sensor was developed and tests were performed to validate this sensor. Based on laboratory tests, it was possible to characterize the proposed current sensor with respect to the number of turns and cross-sectional area of the primary and secondary coils. Furthermore, using the Least Squares Method, it was possible to determine the efficiency of the air core transformer current sensor (the best efficiency found, considering different test conditions, was 2%), which leads to a linear output response.

## 1. Introduction

The international concern about the shortage of environmental resources dates from 1970, when international conventions took place with the goal of slowing down natural resource consumption [1]. Among these conventions, one can cite the United Nations Conference on the Human Environment that occurred in Stockholm in 1972. Later, other conferences took place around the world with the same objective as, for example, the Vienna Convention for Ozone Layer Protection in 1985, the Montreal Protocol in 1987, the United Nations Conference on Environment and Development—ECO’92 in 1992, the United Nations Conference in 1996 and the Kyoto Protocol in 1997.

The concept of “sustainable development” was firstly introduced in 1987 by the UN (United Nations) and emphasized that all development must respond to actual demands without shorting future generations’ capability of satisfying their own necessities [1,2,3]. This concept was directly applied to the construction industry, as defined in the Second United Nations Conference on Human Settlements (Habitat II) held in Istanbul in 1996.Since this conference, the European construction industry began considering the concept of sustainable development for buildings and some regulations and standards emerged in France(RT 2000), Switzerland (Minergie), and Germany (Habitat Passivo) [4,5].

The European Commission (EC) adopted, in 2006, a plan with the objective of reducing energy consumption in 20% by 2020. This plan is called “Action Plan for Energy Efficiency” and is supported by directives. In this context, the EPDB (Directive of Energy Performance of Building) enabled the development of some standards regarding energy calculation methods in buildings. An example of these standards is the standard EN 15232 “Energy Performance of Buildings—Impact of Building Automation, Control, and Building Management” [6], which presents methodologies for assessing/calculating the influence of building automation and of technical building management on the buildings’ energy consumption. Besides, the standard ISO 50001 “Energy Management Standard” permits organizations (as industrial plants, institutional, commercial, and governmental facilities) to establish the requirements and systems that are necessary to improve energy performance [7].

Nowadays, many countries are producing standards and regulations aiming for the application of the sustainable development concept to buildings. Some of these countries (for example, France, Germany, Switzerland, Japan, Mexico, Australia, USA, and Canada) already have their own environmental certification systems that consider some sustainability criteria. Among these certification systems, one can cite: LEED (Leadership in Energy and Environmental Design, from USA) [8,9,10], BREEAM and ECOHOMES (BRE Environmental Assessment Method, from the United Kingdom) [10,11,12], CASBEE (Comprehensive Assessment System for Building Environmental Efficiency, from Japan) [10,13,14], HQE (Haute Qualité Environnementale dês Batiments, from France) [15] and GREEN STAR (from Australia) [10,16].

In Brazil, the concept of sustainable development is also applied to civil industry using building certification systems. These systems, which are the focus of this work, correspond to LEED and to AQUA (High Environmental Quality) which is based on HQE from France [17].

Furthermore, since 1970, the world energy industry has been changing due to the oil crises and to environmental pressures. Thus, in recent decades, there was a great incentive to use renewable energy sources and also to draft laws with the objective of protecting the environment, besides the launching of programs that encourage the reduction of electricity consumption. Regarding the Brazilian construction sector, there was, in 2001, the development and the regulation of Law number 10.295 [18], determining the creation of mechanisms for the development of more efficient buildings (from the energy consumption/generation point of view).After years of work involving various institutions, a standard for energy efficiency in buildings was regulated. This standard corresponds to the regulations for voluntary labeling of energy efficiency levels in commercial, service, and public buildings. This regulation classifies buildings based on their energy efficiency level and is founded on three main requirements, according to a government program named Procel Edifica. These requirements are: efficiency and installed capacity of the lighting system; efficiency of the air-conditioning system, and thermal performance of the building envelope [18,19].

Both LEED and AQUA certifications as well as Procel Edifica labeling process embolden the implementation of initiatives aiming to increase energy efficiency in buildings to be built and their operation. Therefore, the use of renewable energy sources, such as wind power and solar energy (photovoltaic panels and solar collectors) and the use of automation (in order to reduce the electric energy consumption) are encouraged. Moreover, the use of cogeneration systems and technical innovations or systems (as natural lighting, for example) is also promoted [9,10,18].

In these certification systems, increased energy efficiency arising from such initiatives must be demonstrated (proved) so that the building in question can receive the score points (related to these initiatives) scheduled in these certification systems. As an example, for Procel Edifica program, the heating water must prove compliance to 70% of solar fraction; wind or photovoltaic energy should provide a minimum savings of 10% in annual energy consumption; and the cogeneration and the technical innovations or systems that increase the energy efficiency of building systems must have a minimum savings of 30% of annual electricity consumption [18,19].

Currently, this verification is carried out using simulation software, simulation methods, and forecasting methods for the different environmental certification systems [20,21,22,23,24,25,26]. In this context, reference [20] presents calculation methods that are able to predict energy savings in residential buildings; and reference [23] presents regression models in order to predict the heating demand for residential buildings on a monthly basis. In [24], the authors use different heat rejection methods to predict the energy consumption of air-conditioning systems and reference [25] uses an artificial neural network to predict the energy in buildings. Reference [26] estimates energy profiles of buildings with similar characteristics using the software EnergyPlus and reference [12] presents a case study regarding building energy simulation tools and their impact on ratings of the environmental certification systems BREEAM and LEED. In [27], the authors present case studies using the EnergyPlus simulation software to estimate potential savings and energy demands in European office buildings. In this regard, other simulation software options are also widely used, such as DOE-2, Energy10, Micropas6, and EnergyPro, among others [28,29,30].

These methods have as a common characteristic the fact that they are based on models and approximate representations of the actual conditions of the building. Consequently, the results provided by them can lead to invalid conclusions regarding evidence of increased energy efficiency.

Thus, a challenge to be faced is to prove, by measuring, the actual increase on the building’s energy efficiency that arises from these initiatives. More specifically, the problem to be dealt with is how to quantify the increase in energy efficiency related to each of the employed initiatives. For instance, in the example cited before, the problem is how to measure if the installed photovoltaic panels provided a minimum savings of 10% in annual energy consumption, or how to measure that the lighting automation system (and the consequent use of natural light) caused a minimum savings of 30% of the annual electricity consumption.

Within this context, there are also methodologies that address the optimization of energy consumption in buildings [31], considering data measured in the buildings. Regarding that, integrated building automation systems can be used, for example, to control air-conditioning (HVAC), ventilation, heating, and lighting systems [31,32]. As an example, reference [32] presents an integrated system for the simulation and optimization of energy consumption in buildings (industrial, residential, public and commercial buildings), intending to enhance the interactivity of building automation systems. The described integrated software tool provides control and automation of different systems (heating, cooling, lighting, air conditioning, and ventilation) aiming to increase energy efficiency according to the energy efficiency requirements of EN 15232 and ISO 50001 [6,7].

It is important to emphasize that the simulation tool proposed in [32] uses data directly measured in the building, including the electrical quantities. However, the common devices adopted to measure these values usually require physical intervention on the building’s electrical installation. This means that, in order to apply this methodology to a building already in operation, it is necessary to physically cut off the circuit conductors under analysis, resulting in the need for reforms.

In this context, in order to properly quantify the increase on energy efficiency that is directly related to the previously mentioned initiatives, it is necessary (a) to measure the energy consumption (or power generation, if applicable) related to each of the employed initiatives, in an easy, efficient way and at low cost implementation; and (b) to take into account that for buildings that are already in operation, the measurements that need to be carried out must be made causing the least possible impact on the building, avoiding significant reforms and consequent financial costs. In this context, this work aims to present a non-destructive method for current sensing in low voltage circuits that allows the evaluation of power consumption/generation in the distributed circuits along the building, based on data that are directly measured from the circuits under analysis. Thus, it makes it possible to assess in detail the initiatives implemented to increase energy efficiency in buildings with environmental certification without retrofitting and with low costs.

This paper is organized as follows. Section 2 describes the non-destructive method and the current sensor proposed in this work, besides the tests that were carried out to characterize the proposed sensor. Section 3 presents the results of this characterization tests and also of the amplifying/filtering stage. Finally, Section 4 presents the main conclusions of this work.

## 2. Experimental Section

The following sections present the non-destructive method, the sensor proposed in this work and the tests carried out aiming the sensor’s characterization.

### 2.1. Power and Energy Evaluation

As previously mentioned, this paper presents a non-destructive method to evaluate initiatives to increase energy efficiency in buildings with environmental certification. In this context, this method proposes the use of a sensor that can be installed directly in the electrical circuit conductors that are powering the initiative in question, without the need for reforms that result in costs and maintenance.

It is assumed that by knowing (a) the current flowing in the circuit under consideration (measured using the sensor proposed here); and (b) the terminal voltage of this circuit (which is known since it corresponds to the circuit nominal voltage), it is possible to determine the circuit’s energy consumption/generation by using Equations (1) and (2) [33]:

(1) P=V×I

(2) $$E=\int_{t1}^{t2}P(t)dt$$

where *P* is the power demanded by the circuit, measured in Watts [W]; *V* is the circuit voltage, measured in Volts [V] (that is known), and *I* is the circuit current, measured in Amperes [A], which is measured with the aid of the proposed sensor and the proposed non-destructive method; *E* corresponds to the energy consumption/generation of the circuit under analysis, from time *t*1 to time *t*2, in Watts x hours [W × h].

Based on the calculated circuit energy consumption/generation, one can determine whether the initiative for increasing energy efficiency achieved its goals. Using the examples previously cited, for Procel Edifica program, from the calculation of this power generation, one can assess whether the implementation of the photovoltaic generation system resulted in savings of at least 10% in the building annual energy consumption; or from the determination of the lighting circuit (or circuits) power consumption, one can check if there was a decrease of at least 30% of electricity consumption associated to lighting, due to the implementation of lighting automation systems (and consequent increased use of natural light).It is important to emphasize that the objective of this work is not to verify the efficiency of the implementation of these initiatives, but to present the non-destructive method and the sensor to be used for this purpose.

The following item presents the sensor used for measuring the current flowing in the circuit to be analyzed in the context of the non-destructive method.

### 2.2. Proposed Sensor

**Figure 1:**
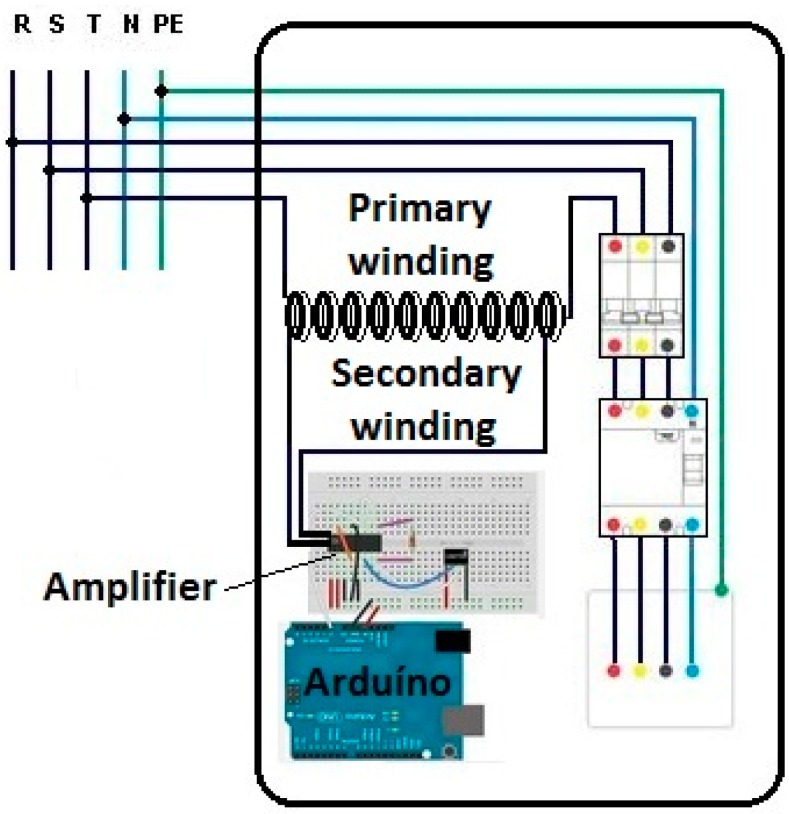
Proposed sensor installed within the switchboard of a building.

The proposed sensor consists of three elements: a current sensor that corresponds to a transformer with an air core with concentric primary and secondary coils, using the low voltage conductors already installed in the building without interrupting or cutting them; a low cost electronic amplifying/filtering stage; and a general use microprocessor (Arduino) [34], as can be seen in Figure 1.

#### 2.2.1. Air-Core Transformer

The proposed current sensor is an air-core transformer [35] with concentric primary and secondary coils (concentric solenoids). Air-core transformers are normally used at radio frequencies, when iron core losses are too high. For lower frequencies, other core materials are preferred as, for example, powdered iron or ferrite [36,37]. Despite the fact that the proposed sensor was developed to be used in low-frequency applications (electric power distribution systems that operate at 50 Hz or 60 Hz), its core is constituted of air. This can be explained by the fact that the proposed sensor is used within a non-destructive method. The idea is that the monitoring of the current of a circuit (and the subsequent calculation of the energy consumed or generated by it) can be done without the need for reforms in the building, directly in the building switchboard or inside power plug boxes, without physically cutting the circuit under analysis. In this context, the primary coil of the transformer is formed with the same conductor of the circuit under analysis. Thus, the use of an air-core transformer as a current sensor is justified by the fact that the use of a ferromagnetic core (commonly adopted in low frequency applications) would not enable the installation of the sensor without physical intervention on the building switchboard or plug boxes.

**Figure 2:**
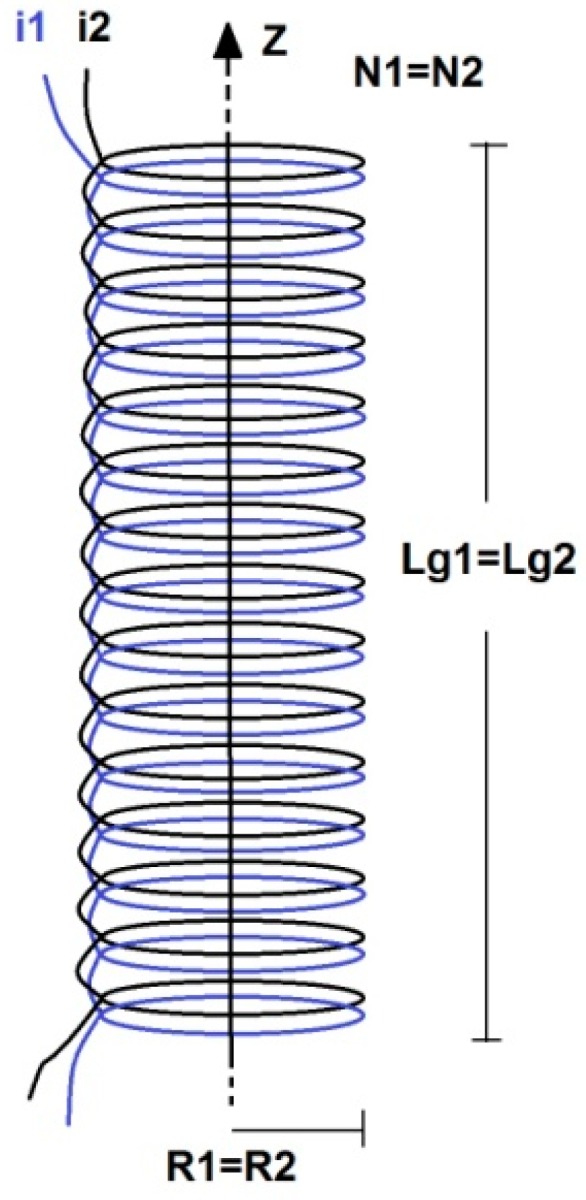
Concentric solenoids.

In this work, the primary and the secondary coils have the same radius (*R*_1_ = *R*_2_), the same length (*Lg*_1_ = *Lg*_2_) and the same number of turns (*N*_1_ = *N*_2_), as illustrated in Figure 2.It is important to emphasize that the primary number of turns is equal to the secondary number of turns, and in this work, this number varies according to the test performed (5, 10, and 15 turns).

One of the conductors of the circuit under consideration corresponds to the primary coil of the transformer. The transformer secondary coil is wound with the primary coil (so that both coils are concentric) within the switchboard of the electrical installation of the building, as previously shown in Figure 1.

The current of the circuit under analysis flows in the primary of the transformer (*I_f_*) and induces a current in the secondary coil of the transformer (*I_i_*) which is then measured. Since there is a mathematical relation between these two currents, it is possible to determine *I_f_* based on *I_i_*.

In an ideal transformer, the magnetic flux produced by the secondary winding of the transformer is given by

(3) $$\phi_{2}=\left(M_{12}\times I_{1}+L_{2.}\times I_{2}\right)$$

where ϕ_2_ is the magnetic flux produced by the secondary of the transformer, *M*_12_ is the transformer mutual inductance, *L*_2_ is the inductance of the secondary, *I*_1_ e *I*_2_ are, respectively, the primary and the secondary currents.

**Figure 3:**
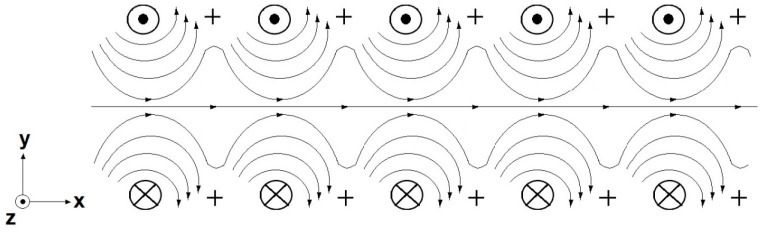
Magnetic flux that is not concatenated by the transformer secondary coil.

In this work, due to the adopted air-core transformer specific geometry, the magnetic flux generated in the primary coil is not fully concatenated by the transformer secondary coil, having a significant impact in the results, as shown in Figure 3. In this figure, the circle with an inner dot represents the primary coil current “coming out” of the x-y Cartesian plane and the circle with an inside cross represents the primary coil current “entering” the x-y Cartesian plane. The crosses represent the conductors of the secondary coil. The arrows indicate the magnetic flux produced by the primary coil. It is possible to observe that a significant part of the generated magnetic flux does not trespass the secondary turns and, consequently, is not concatenated by the secondary coil.

Considering this situation, where the transformer is not ideal, Equation (3) can be rewritten as [34]

(4) $$\phi_{2}=\eta \times \left(M_{12}\times I_{1}+L_{2.}\times I_{2}\right)$$

where η can be understood as the efficiency of the air-core transformer.

The voltage at the transformer secondary is given by

(5) $$V_{2}=\frac{d{\text{ϕ}}_{2}}{dt}$$

For the secondary under a short-circuit condition (situation in which the tests were carried out in this work, as detailed in Section 3), it is possible to write

(6) $$V_{2}=\frac{d{\text{ϕ}}_{2}}{dt}=0$$

Thus

(7) $$\frac{d\phi_{2}}{dt}=\frac{d[\eta \times \left(M_{12}\times I_{1}+L_{2.}\times I_{2}\right)]}{dt}=0$$

or

(8) $$-\eta \times M_{12}\times \frac{dI_{1}}{dt}=L_{2}\times \frac{dI_{2}}{dt}$$

The currents *I*_1_ and *I*_2_ correspond to sinusoids, according to Equations (9) and (10) respectively

(9) $$I_{1}\left(t\right)=A\times sen\left(wt+\varphi \right)$$

(10) $$I_{2}\left(t\right)=B\times sen\left(wt+\delta \right)$$

where *A* and *B* correspond to the amplitudes of the currents *I*_1_ and *I*_2_, respectively; φ and δ correspond to the angular phase shift of currents *I*_1_ and *I*_2_ respectively; *w* is the angular frequency which is a constant and is equal to (2πf) and f corresponds to the electric power system operating frequency (in the case of Brazil, 60 Hz).

So

(11) $$\frac{dI_{1}\left(t\right)}{dt}=A\times w\times \cos\left(wt+\varphi \right)$$

(12) $$\frac{dI_{2}\left(t\right)}{dt}=B\times w\times \cos\left(wt+\delta \right)$$

Equation (8) can be rewritten, in module, as

(13) $$\left|-\eta \times M_{12}\times \frac{dI_{1}}{dt}\right|=\left|L_{2}\times \frac{dI_{2}}{dt}\right|$$

or

(14) $$\eta \times M_{12}\times \left|\frac{dI_{1}}{dt}\right|=L_{2}\times \left|\frac{dI_{2}}{dt}\right|$$

But

(15) $$\left|\frac{dI_{1}}{dt}\right|=\frac{A\times w}{\sqrt{2}}$$

(16) $$\left|\frac{dI_{2}}{dt}\right|=\frac{B\times w}{\sqrt{2}}$$

Consequently

(17) $$\eta \times M_{12}\times \frac{A\times w}{\sqrt{2}}=L_{2}\times \frac{B\times w}{\sqrt{2}}$$

Thus

(18) $$B=\frac{\eta \times M_{12}\times A}{L_{2}}$$

But

(19) $$L_{2}=\frac{\mu_{0}\times N_{2}^{2}\times \pi \times R_{2}^{2}}{Lg_{2}}$$

where μ_0_ is the magnetic permeability of vacuum (adopted as the magnetic permeability of air); *N*_2_ is the number of secondary windings; *R*_2_ is the radius of the secondary coil and *Lg*_2_ is the length of the secondary coil [34].

The mutual inductance can be expressed by:

(20) $$M_{12}=\frac{\mu_{0}\times N_{1}\times N_{2}\times \pi \times R_{1}^{2}}{Lg_{1}}$$

As previously mentioned, in this work *N*_1_ = *N*_2_, *R*_1_ = *R*_2_ and *Lg*_1_ = *Lg*_2_.Thus

(21) $$M_{12}=\frac{\mu_{0}\times N_{2}^{2}\times \pi \times R_{2}^{2}}{Lg_{2}}=L_{2}$$

Therefore, Equation (18) can be rewritten as

(22) $$B=\frac{\eta \times L_{2}\times A}{L_{2}}=\eta \times A$$

In this work, the magnitude of the secondary induced current can be written as

(23) $$\left|I_{2}\right|=\left|I_{i}\right|=\frac{B}{\sqrt{2}}$$

and the magnitude of the current flowing in the primary can be written as

(24) $$\left|I_{1}\right|=\left|I_{f}\right|=\frac{A}{\sqrt{2}}$$

Thus, Equation (22) can be rewritten as

(25) $$\left|I_{i}\right|=\eta \times \left|I_{f}\right|$$

This means that the relation between the current measured by the proposed sensor (*I_i_*) and the current flowing in the circuit under analysis (*I_f_*) can be defined by as a straight line, given by the well-known equation:

(26) y=a×x

where *y* and *x* correspond respectively to the output (*I_i_*) and input (*I_f_*) of the system under analysis; and the parameter “*a*”, which is the slope of the line, is the transformer efficiency (η), to be determined.

The transformer efficiency (η) can be determined from the application of the Least Squares Method (LSM), widely spread in the literature [38]. In this method, the inputs and outputs of the system under analysis are represented as vectors, and the parameter of the model can be determined using Equation (27)

(27) $$\eta ={\left[{\left({\hat{I}}_{f}\right)}^{T}\times \left({\hat{I}}_{f}\right)\right]}^{-1}\times {\left({\hat{I}}_{f}\right)}^{T}\times \left({\hat{I}}_{i}\right)$$

Thus, by determining the value of η (using Equation (27)) and measuring *I_i_* (using the proposed sensor), it is possible to determine the current flowing in the circuit under analysis (*I_f_*), using Equation (25).

Finally, after the measurement of *I_i_* is performed, it can be amplified, filtered and transmitted to a microprocessor where the AC circuit’s consumed or generated energy can be calculated. The steps of amplification/filtering, and the microcontroller use are described in the following items.

#### 2.2.2. Amplifying/Filtering Stage

**Figure 4:**
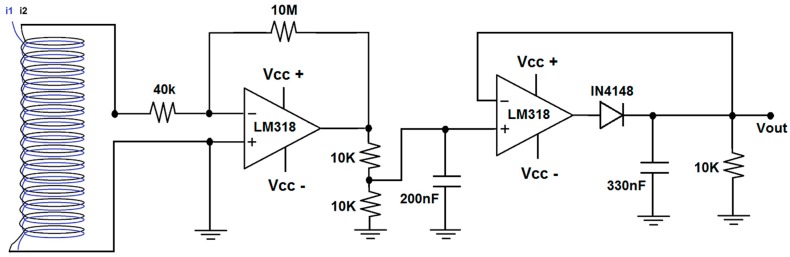
Electronic circuit of the amplifying/filtering stage connected to the current sensor (air-core transformer).

The current *I_i_* that is measured in the secondary coil of the transformer is, then, amplified and filtered with the aid of an electronic circuit that is illustrated in Figure 4.This circuit has an amplifier stage and a filtering stage that use the LM318 operational amplifier. The expected output thus varies in the range of 0–3 V for input currents in the range of 0–30 A.

#### 2.2.3. Microcontroller

**Figure 5:**
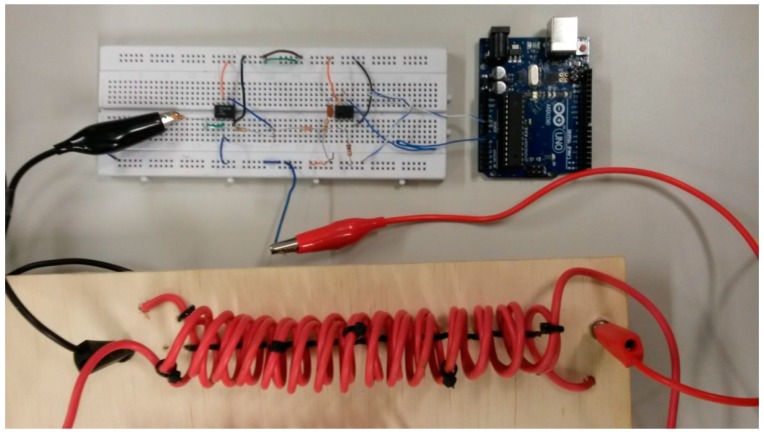
Prototype of the proposed sensor.

The microcontroller board used in this work is the Arduino Uno, which is based on ATmega328 microcontroller. It has 14 digital input/output pins and six analog inputs [39]. This board is able to process the information related to the measured values of *I_i_* that come from the amplifying/filtering stage and can determine the energy consumption/generation, according to Equations (1) and (2). Figure 5 illustrates the prototype of the complete experimental sensor, integrating air core transformer coils, electronic circuit and microcontroller board.

### 2.3. Laboratory Tests Description

**Figure 6:**
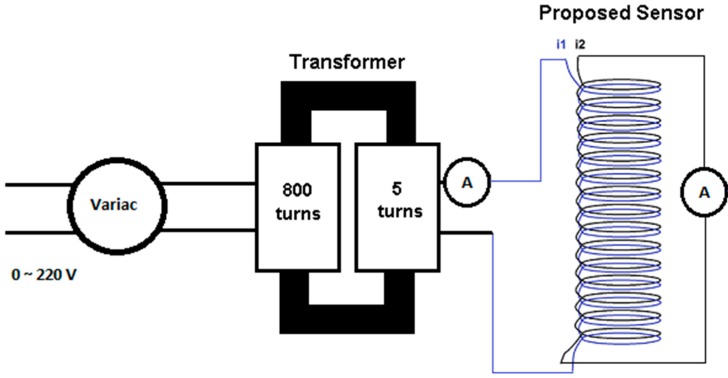
Electrical diagram of the test bench used in the current sensor characterization tests.

Laboratory tests were carried out with the objective of characterizing the proposed current sensor (air-core transformer with concentric secondary and primary coils). Figure 6 illustrates the test bench used to characterize the sensor.

In this figure, the conductor that energizes the circuit under analysis (that must prove an increase of energy efficiency) is the primary coil of the transformer (that forms the proposed sensor). Consequently, as described in the previous section, the current flowing in the circuit under consideration (*I_f_*) is the current flowing through the transformer primary coil. The current flowing through the secondary of the transformer is the current that is induced in the secondary coil (*I_i_*) and which is effectively measured by the proposed sensor. The primary of the transformer is fed by a source of alternating voltage (Variac) connected to another power transformer (with turns ratio of 800:5 or 160:1)which allows the primary current *I_f_* to assume *a priori* established values (measured with the aid of an ammeter).In this test, *I_f_* values varied between 1 A and 30 A. For each value of *I_f_*, the corresponding value of *I_i_* was measured (in the transformer secondary that was short-circuited) with the aid of another ammeter.

This test was first conducted with the coils of the primary and secondary of the proposed sensor consisting of a copper conductor with cross-sectional area of 2.5 mm^2^.The coils were formed with 5, 10, and 15 turns. Subsequently, this procedure was repeated for copper conductors with cross-sectional areas of 4 mm^2^ and 6 mm^2^.

Afterwards, the complete experimental sensor was tested, in order to verify its output voltage response as the primary coil current varied. The results of all tests are described in Section 3.

## 3. Results and Discussion

**Figure 7:**
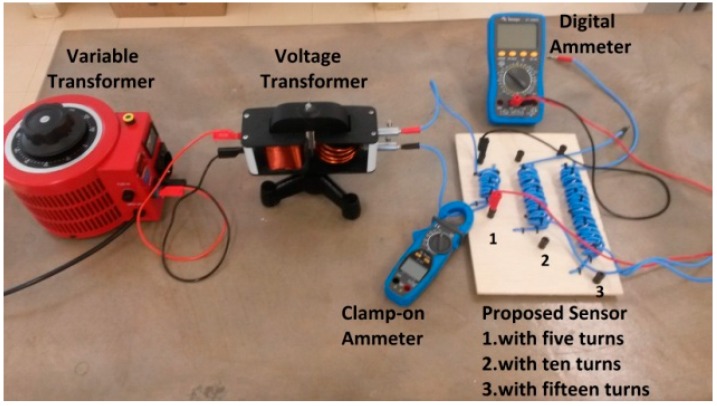
Test bench for the sensor characterization.

As previously mentioned, the proposed current sensor consists of two concentric solenoids with equal radius (*R*_1_ = *R*_2_), equal length (*Lg*_1_ = *Lg*_2_) and equal number of turns (*N*_1_ = *N*_2_), as illustrated in Figure 2. In this work, tests were carried out considering conductors with different cross-sectional areas. The characteristics of the concentric solenoids for conductors of 2.5 mm^2^, 4 mm^2^, and 6 mm^2^are presented in Table 1. Figure 7 shows the test bench implemented to perform the sensor characterization tests. In this figure, one can observe the proposed sensor (two concentric coils with air core) and the equipment for performing these tests, as previously shown in Figure 6.

**Table 1:** Characteristics of the concentric solenoids for conductors of 2.5 mm^2^, 4 mm^2^ and 6 mm^2^.

	5 Turns	10 Turns	15 Turns
**Internal radius (m)**	0.01	0.01	0.01
**Length (m)**	0.05	0.10	0.15

The results from these tests are presented as follows for conductors with cross-sectional areas of 2.5 mm^2^, 4 mm^2^, and 6 mm^2^, considering situations with number of turns of the primary and secondary equals to 5, 10, and 15.

### 3.1. Influence of Number of Turns

As previously mentioned, the current sensor was developed to be applied in buildings that are already in operation, without the need for reforms and with rapid and low cost implementation. So, the use of this sensor is physically limited by the actual conditions of the switchboard, which already contains other circuit elements (sockets, protection devices, *etc.*). Thus, it was assumed, in this work, that these conditions impose limitations on the maximum and minimum number of turns (of the current sensor) that can be practiced in the field, considering the integration with conventional meters or with the Building Automation System (BAS). Thus, it is important to check the influence of the number of turns in the sensor’s induced current and, in order to reach this goal, this work considers actual installation conditions. Consequently, the influence of the number of turns was tested for a minimum of 5 turns and a maximum of 15 turns. The sensor configuration was also tested with 10 turns to broaden its characterization analysis.

**Figure 8:**
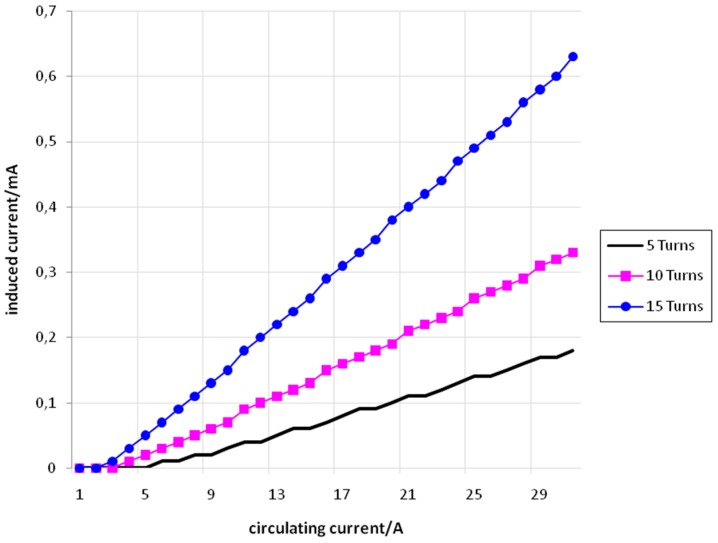
*I_f_ versus I_i_* for the conductor of 2.5 mm^2^ with 5 turns (black), 10 turns (pink), and 15 turns (blue).

Figure 8 shows the values of *I_f_* (set) and *I_i_* (measured) for the conductor of 2.5 mm^2^ with 5, 10, and 15 turns.

**Figure 9:**
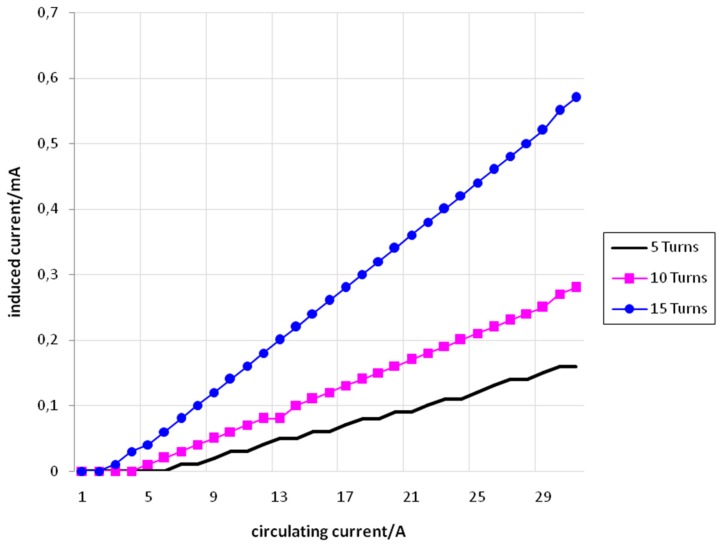
*I_f_ versus I_i_* for the conductor of 4mm^2^with 5 turns (black), 10 turns (pink), and 15 turns (blue).

Figure 9 shows the values of *I_f_* (set) and *I_i_* (measured) for the conductor of 4 mm^2^ with 5, 10, and 15 turns.

**Figure 10:**
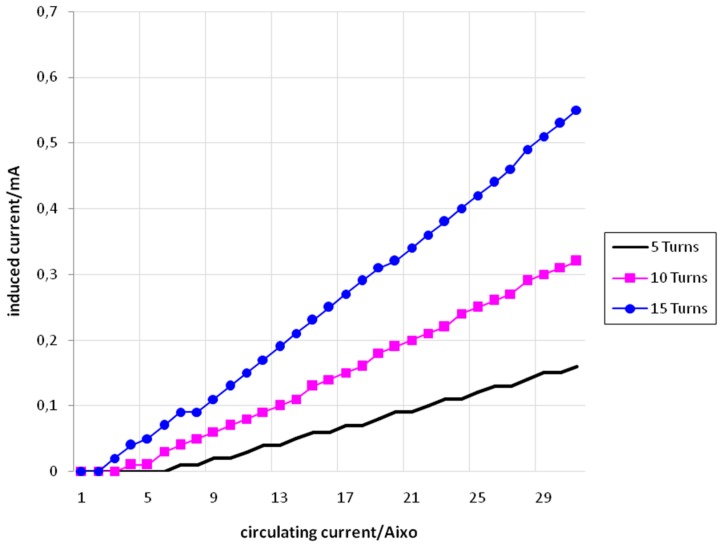
*I_f_ versus I_i_* for the conductor of 6mm^2^with 5 turns (black), 10 turns (pink), and 15 turns (blue).

Figure 10 shows the values of *I_f_* (set) and *I_i_* (measured) for the conductor of 6 mm^2^ with 5, 10, and 15 turns.

From Figure 8, Figure 9 and Figure 10 it is possible to see that the relation between *I_f_* and *I_i_* is approximately linear. It is also possible to conclude that for a same value of *I_f_,* the value of *I_i_* increases according to the increase in the number of turns. This fact can be explained using Equation (28)

(28) $$\phi_{1}=\frac{\mu_{0}\times N_{1}\times I_{f}\times A}{Lg1}$$

where ϕ_1_ is the magnetic flux produced by the primary coil, µ_0_ is the vacuum magnetic permeability (that was considered as the air magnetic permeability), *N*_1_ is the number of turns of the primary coil, *I_f_* is the flowing current (or circulating current) in the primary coil, A is the cross-sectional area of the magnetic perimeter and *Lg*_1_ is the length of the primary coil. The induced current (*I_i_*) in the secondary current is proportional to ϕ_1_. So, if*N*_1_ increases, ϕ_1_ increases and, consequently, *I_i_* increases.

### 3.2. Influence of Conductors Cross-Sectional Area

As discussed in Section 3.1, three base cases were adopted for the tests involving the number of turns (5, 10, and 15 turns), considering the operational conditions of an actual electrical installation. Similarly, to verify the influence of the conductor’s cross-sectional area in the sensor’s induced current, some practical aspects, related to the electrical conductor that forms the sensor’s coils, must be considered.

In Brazil, the conductors that are most commonly used in low voltage electrical installations usually have standard cross-sectional areas of 1.5 mm^2^, 2.5 mm^2^, 4 mm^2^, 6 mm^2^, and 10 mm^2^. Among these, conductors of 1.5 mm^2^ are exclusively employed in lighting circuits. The conductors of 2.5 mm^2^, 4 mm^2^, and 6 mm^2^ represent more than 90% of the conductors used in built environments. Thus, it was considered appropriate to carry out experimentations using coils formed with the most representative cross-sectional areas conductors (2.5 mm^2^, 4 mm^2^, and 6 mm^2^), considering the three different number of turns that were previously adopted (5, 10, and 15 turns).

**Figure 11:**
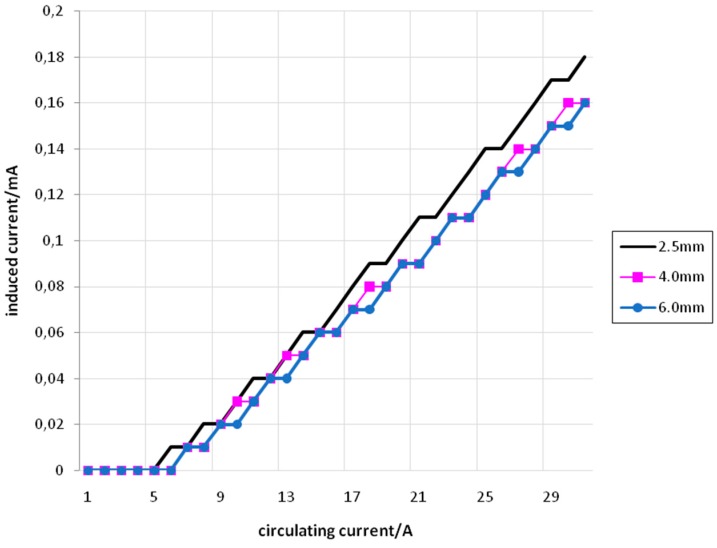
*I_f_ versus I_i_* for 5 turns with conductors of 2.5 mm^2^ (black), 4 mm^2^ (pink) and 6 mm^2^ (blue).

Figure 11 shows the relation between *I_f_* (set) and *I_i_* (measured) for primary and secondary coils with 5 turns, using conductors of 2.5 mm^2^, 4 mm^2^, and 6 mm^2^.

**Figure 12:**
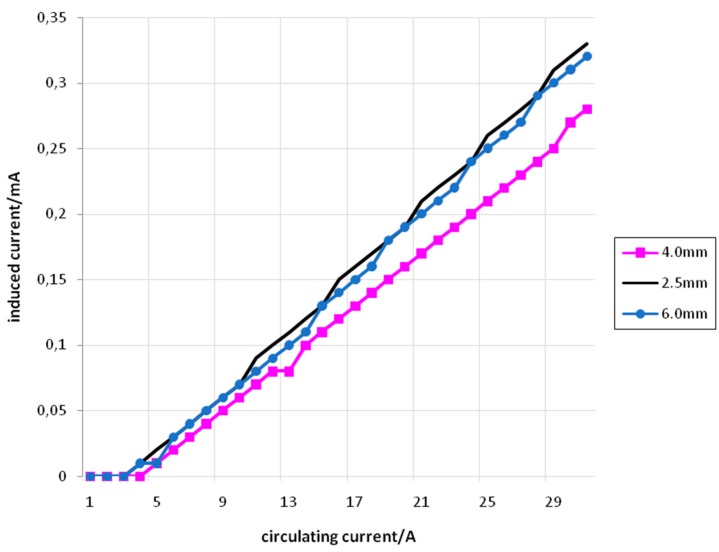
*I_f_ versus I_i_* for 10 turns with conductors of 2.5 mm^2^ (black), 4 mm^2^ (pink), and 6 mm^2^ (blue).

Figure 12 shows the relation between *I_f_* (set) and *I_i_* (measured) for primary and secondary coils with 10 turns, using conductors of 2.5 mm^2^, 4 mm^2^, and 6 mm^2^.

**Figure 13:**
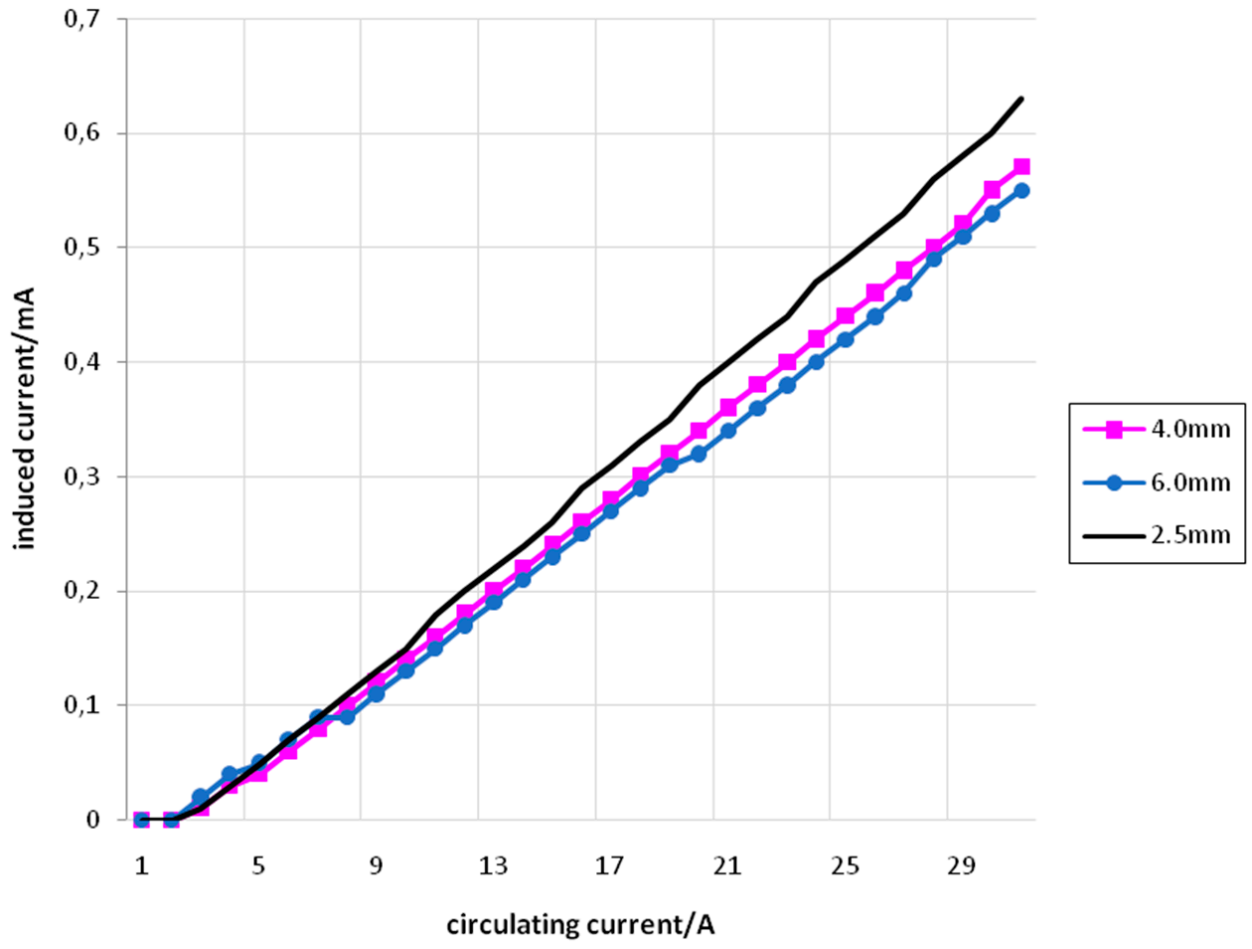
*I_f_ versus I_i_* for 15 turns with conductors of 2.5 mm^2^ (black), 4 mm^2^ (pink), and 6 mm^2^ (blue).

Figure 13 shows the relation between *I_f_* (set) and *I_i_* (measured) for primary and secondary coils with 15 turns, using conductors of 2.5 mm^2^, 4 mm^2^, and 6 mm^2^.

From Figure 11, Figure 12 and Figure 13 it is possible to see that the relation between *I_f_* and *I_i_* is approximately linear. Furthermore, the straight lines obtained for conductors with different cross-sectional areas are very similar. The small difference that can be seen in the angular coefficients of these straight lines can be explained by the differences that appear in the cross-sectional areas associated with the coils. These cross-sectional areas suffer slight reductions as the conductor’s cross-sectional areas increase. Also, other small area differences are inherently associated to the coils’ construction, subjected to practical imperfections.

### 3.3. Air-Core Transformer Efficiency

Using Equation (27), it was possible to determine the air-core transformer efficiency for the different test conditions used in this work, as presented in Table 2.

**Table 2:** Air-core transformer efficiency.

	Conductor of 2.5 mm^2^	Conductor of 4 mm^2^	Conductor of 6 mm^2^
**5 turns**	η = 0.54%	η = 0.49%	η = 0.48%
**10 turns**	η = 1.04%	η = 0.86%	η = 1.01%
**15 turns**	η = 2.00%	η = 1.81%	η = 1.74%

From Table 2, it is possible to see that the best efficiency (2%) is associated with the condition of 15 turns and the conductor of cross-sectional area of 2.5 mm^2^. This low efficiency is due to the significant part of the magnetic flux that is not concatenated by the secondary coil, since the transformer core is constituted by air. Despite this fact, the necessity of using an air-core transformer is justified within the context of the proposed non-destructive method, as previously mentioned.

### 3.4. Mathematical Models

Using Equations (25) and (27), it was also possible to determine a mathematical model to describe the relation between *I_i_* and *I_f_*, as presented in Table 3.

**Table 3:** Mathematical models for *I_i_ versus I_f_*.

	Conductor of 2.5mm^2^	Conductor of 4mm^2^	Conductor of 6mm^2^
**5 turns**	$I_{i}=0.0054\;\times I_{f}$	$I_{i}=0.0049\times I_{f}$	$I_{i}=0.0048\times I_{f}$
**10 turns**	$I_{i}=0.0104\times I_{f}$	$I_{i}=0.0086\times I_{f}$	$I_{i}=0.0101\times I_{f}$
**15 turns**	$I_{i}=0.02\times I_{f}$	$I_{i}=0.0181\times I_{f}$	$I_{i}=0.0174\times I_{f}$

### 3.5. Results of the Amplifying/Filtering Stage

**Figure 14:**
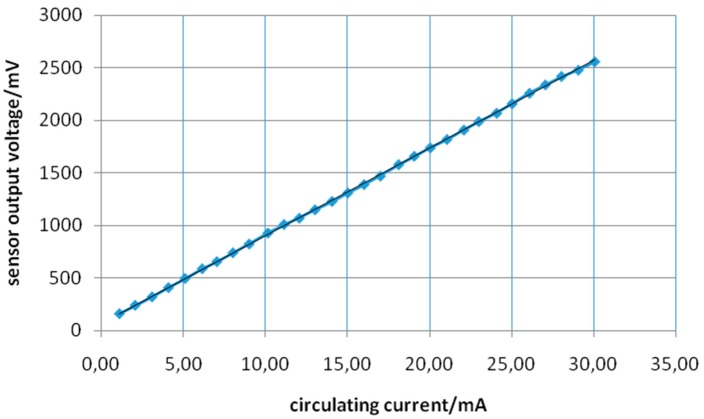
Relation between *I_f_* and the sensor output voltage after the amplifying/filtering stage.

Figure 14 shows the relation between *I_f_* and the sensor output voltage after the amplifying/filtering stage.

From Figure 14 it is possible to observe that the relation between the complete sensor output voltage and the input current (that corresponds to the current under analysis, *I_f_*) is linear and varies in the range of 0–3 V. This voltage range permits the usage of a broad family of analog-digital converters and microcontrollers and, consequently, enhances the applicability of the proposed transducer and amplifier/filter associated circuits in a wide range of sensors networks platforms, with easy embedded software implementation of its linear response.

### 3.6. Discussion

The method proposed in this paper corresponds to a non-destructive current sensing approach for the assessment of initiatives to increase energy efficiency in buildings with environmental certification. More specifically, this method allows the quantitative evidence of reducing or increasing the power consumption of circuits that power initiatives to increase energy efficiency in buildings.

Nowadays, this verification is carried out, for different environmental certification systems, using software and simulation methods, as presented by references [20,21,22]. Since the software and simulation methods are based on models and/or approximated and hypothetical representations of the building’s actual conditions, the results provided by them may not be in fact what is really happening in the building. In other words, even if the software or the simulation method used is precise, it will provide only estimated results and may lead to inappropriate conclusions regarding the evidence of increased energy efficiency associated to the initiatives under analysis.

There are also methodologies that aim at estimating or predicting the energy consumption of a building as a whole, or of specific systems, as the heating system or the air-conditioning system. According to references [23,24,25,26], these estimates and predictions can be made, for example, based on data extracted from bills or from the systems’ characteristics. These methods also use models to represent global building energy consumption and, therefore, can provide an approximate estimate of power demand that can also lead to invalid conclusions regarding the evidence of increased energy efficiency.

There are also other works that make use of electrical quantities that are measured to carry out the control and automation of different building systems (air conditioning, ventilation, lighting, heating, *etc.*), aimed at increasing the energy efficiency of the building, as described in [32]. However, these electrical parameters are measured by devices that require cutting the circuit under analysis, generating the need for retrofitting, maintenance and, therefore, additional operational costs.

In this context, the proposed method has the advantage of, being based on actual measurements, being able to provide a truly valid proof and quantification of increased energy efficiency of the initiative under consideration. Furthermore, it is also a non-destructive method, since the current demanded by the circuit under analysis is measured without the need for circuit cutting and retrofitting.

Another strong point of the proposed method consists of the fact that the developed sensor can be attached to a microcontroller, which is able to process the information related to the measured values of I_i_ that come from the amplifying/filtering stage, and, therefore, can automatically determine the energy consumption/generation, according to Equations (1) and (2). Furthermore, as mentioned before, the amplification and filtering stage inserted into the sensor permits the usage of a broad family of analog-digital converters and microcontrollers, broadening the applicability options of the proposed sensor.

On the other hand, it is important to note that a sensitive point of the proposed method corresponds to the efficiency of the air-core transformer used as current sensor. As shown by the obtained results, this efficiency was about 2% at most, considering all tests performed. As previously mentioned, this low efficiency is due to the use of the air core (that makes a significant part of the magnetic flux not to be concatenated by the secondary coil), which is of fundamental importance, considering the non-destructive feature of the proposed methodology. Due to this low efficiency, the insertion of an amplifying/filtering stage in the developed sensor is necessary in order to amplify and filter the current measured by the current sensor.

Despite the mentioned limitations, it is important to note that the proposed methodology can be used directly in environmental certification systems that require proof of increased energy efficiency of buildings, such as AQUA, LEED, and Procel Edifica certification systems. Thus, the use of the sensor and the developed method can enable a building, under evaluation by an environmental certification system, to receive (or not) the score points associated with the increased energy efficiency related to different initiatives such as, for example, the use of photovoltaic panels for generating electricity or the implementation of a lighting automation system.

The proposed methodology can also be easily adopted in the context of the energy efficiency requirements of EN 15232 and ISO 50001. These requirements include the control and the automation of heating, cooling, ventilation, air-conditioning, shading, and lighting systems to enhance energy efficiency in buildings or organizations. In order to really measure and quantify the impact of automation and control systems in the energy efficiency of the building/organization, it is necessary to measure the power consumption of the building. Usually, the measurement of energy consumption associated with different circuits of the building is done using devices that require the physical cutting of the circuit under analysis [32] and, therefore, require reforms in the building. In this sense, the proposed methodology can be used to measure this energy consumption in a low-cost and non-destructive way.

## 4. Conclusions

This paper presented a non-destructive current sensing method for the evaluation of initiatives aimed at increasing energy efficiency in buildings with environmental certification. This method is based on the implementation of a sensor consisting of three elements: current sensor using a transformer with an air core, amplification/filtering stage, and microcontroller. It uses the low-voltage AC circuit wires as the transformer’s coils, avoiding retrofitting and allowing distributed current sensing inside buildings in a clean, low-cost way. Consequently, it has the potential to enhance the dissemination of energy efficiency building certification best practices.

In this work, a prototype was developed and tests were performed to validate the proposed sensor. Based on laboratory tests, it was possible to characterize the current sensor with respect to the number of turns and cross-sectional area of the primary and secondary coils. Considering the influence of the number of turns, it was possible to observe, from the obtained results, that the relation between *I_f_* and *I_i_* is approximately linear. It was also possible to conclude that for a same value of I_f_, the value of *I_i_* increases according to the increase in the number of turns. Besides, regarding the influence of the cross-sectional area of the conductors, it was possible to observe that the relation between *I_f_* and *I_i_* is also approximately linear. Consequently, the straight lines obtained for conductors with different cross-sectional areas are very similar. The small differences found can be explained by the differences that appear in the cross-sectional areas associated with the coils, since these cross-sectional areas suffer slight reductions as the conductor’s cross-sectional areas increase.

Furthermore, using the Least Squares Method, it was possible to determine the efficiency of the air core transformer current sensor and mathematical models that describe the relation between the monitored current *I_f_* and the transducer current *I_i_*. Thus, based on the estimated parameters, it is possible to accurately determine the current demanded by the circuit under analysis, considering a given current value detected in the secondary of the transformer. That result will be later used by the microcontroller to calculate the consumption/power generation associated with the circuit.

Due to the proposed current sensor low efficiency (2% in the best test condition), it became necessary to implement an amplifying/filtering stage. So, the values measured by the current sensor can be used in a microcontroller mainly responsible for consumed/generated energy determination, considering the circuit under analysis.

Regarding the added value of the proposed method, it can be concluded that, unlike other methods widespread in the literature (which are based on software or simulation methods), the sensor and the methodology developed in this work can yield proofs of increased energy efficiency of a building, based on actual measurements. Moreover, this confirmation can be made in a simple and non-destructive way, without the need for additional reforms and costs.

As a future work, it is possible to highlight the development of other current sensors that can maintain the non-destructive characteristic of the proposed method, but that can also provide greater efficiency in the current measurement, with low constructive complexity for operational purposes. For example, the usage of iron core coils can improve the efficiency, but can be hard to implement in the actual switchboard conditions. Moreover, the possibility of using a microcontroller based on wireless technology, for determining the electric energy consumption/generation of the initiative under analysis, can be investigated. Finally, it is important to emphasize that the proposed methodology will be actually applied, in a future work, for the evaluation of initiatives to increase energy efficiency in real cases of Brazilian buildings that are in an environmental certification process.
